# Small Bowel Obstruction Due to Enterolith in a Patient with Diffuse Jejuno-Ileal Diverticulosis

**DOI:** 10.5334/jbsr.1456

**Published:** 2018-01-31

**Authors:** H. Fourneau, B. Coulier, A. Afshin Rezazadeh

**Affiliations:** 1 Department of Radiology, Clinique St. Pierre, Ottignies, BE; 2 Department of Radiology, Clinique St. Luc, Bouge (Namur), BE

**Keywords:** Intestinal diverticula-complications, Jejunal diverticulitis, Enterolith, Intestinal obstruction

## Abstract

We report an unusual case of small bowel obstruction caused by a large enterolith released from small bowel diverticulitis in a 81-year-old patient with occult massive Diffuse Jejuno-Ileal Diverticulosis (DJID). DJID is a rare condition whose symptoms are usually absent or non-specific. In most cases, the diagnosis of DJID is incidentally made or consecutive to secondary complications comprising obstruction, haemorrhage, diverticulitis and perforation. We shortly review the clinical and imaging features and complications of DJID.

## Case Report

**Figure 1:**
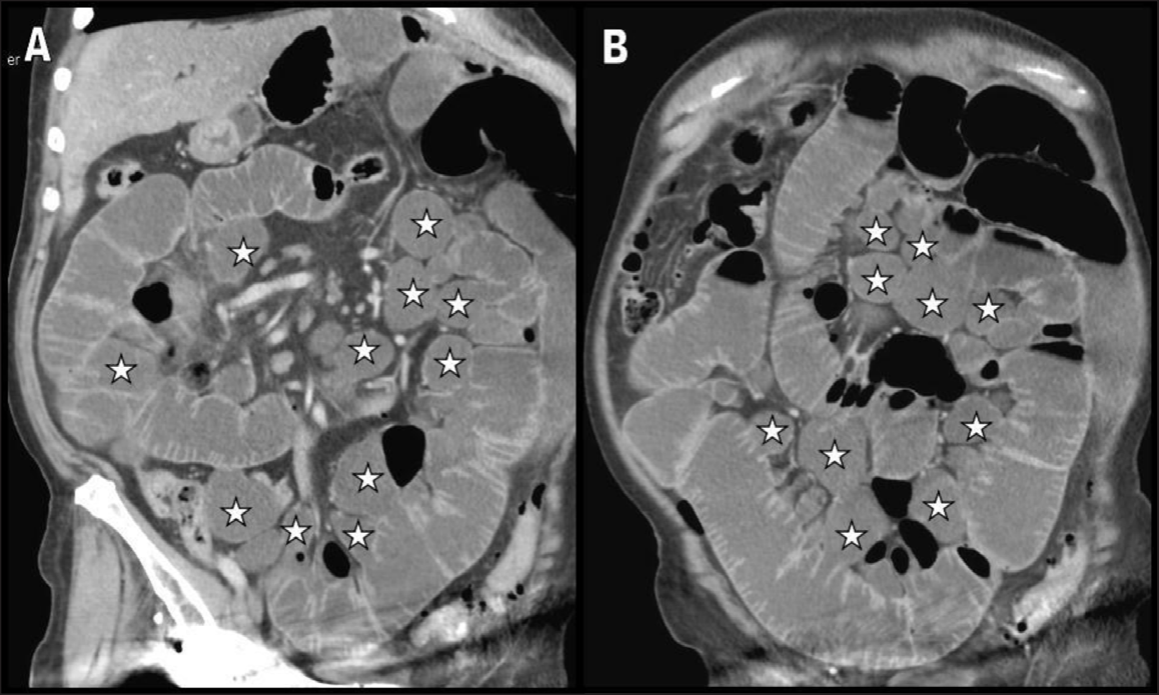
Coronal multiplanar reconstructions of the abdomen (**A** – posterior view and **B** – more anterior view) show massive diffuse fluid distention of the small bowel loops and numerous dilated small bowel diverticula along the mesenteric border (white stars).

**Figure 2:**
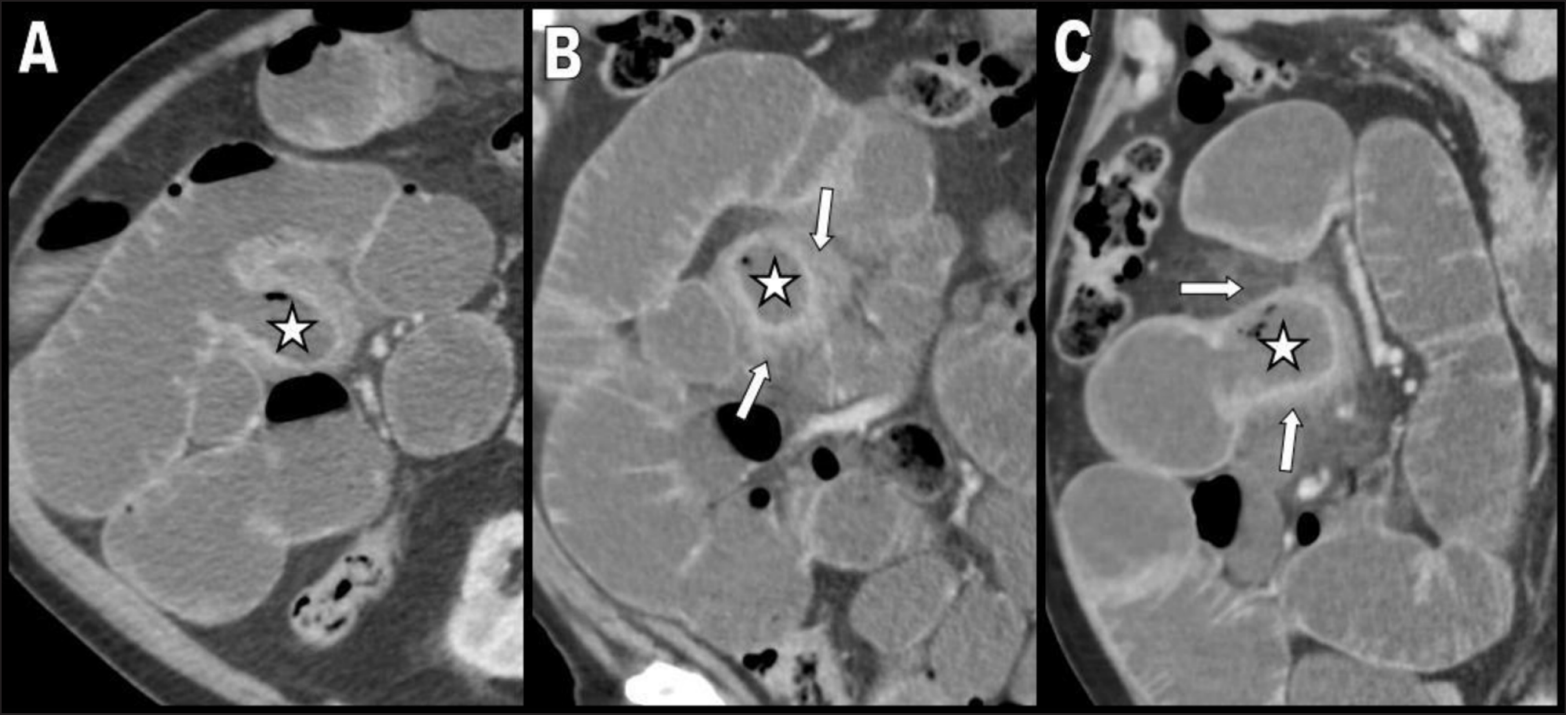
Axial **(A)**, coronal **(B)** and sagittal **(C)** views at the level of the right flank show a distended inflammatory diverticulum (white star) surrounded by inflammatory fat stranding (white arrows).

**Figure 3:**
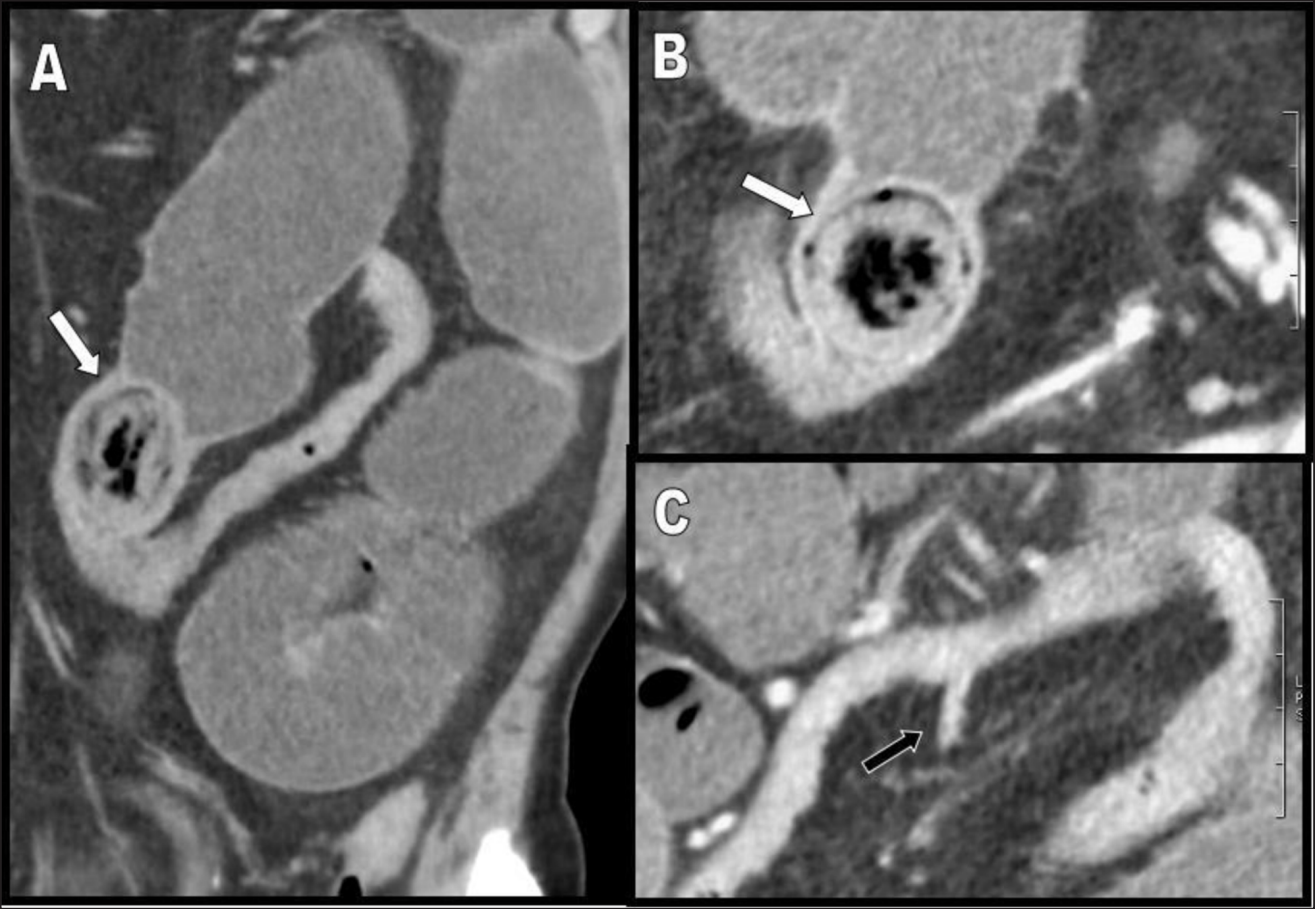
Sagittal oblique **(A)** and axial **(B)** views show a 3cm large stratified enterolith (white arrow) being responsible of the intestinal obstruction in the lower left flank. In the distal ileum, a Meckel’s diverticulum is clearly visible (black arrow on **(C)** and will be used by the surgeon to facilitate the extraction of the enterolith.

An 81-year-old man with an history of Alzheimer’s disease, excess weight and type-2 diabetes presented to the emergency department with a two-day history of diffuse abdominal pain. Contrast-enhanced abdominal computed tomography (CT) showed diffuse fluid distention of the small bowel loops indicative of obstruction. In addition, there was a profusion of dilated small bowel diverticula along the mesenteric border (Figure 1A – coronal posterior and B – more anterior views. White stars indicate the diverticula). An isolated jejunal diverticulitis was diagnosed in the right flank (Figure 2A – axial, B – coronal, and C – sagittal views. White stars indicate the distended inflammatory diverticulum and white arrows show the surrounding inflammatory fat stranding). The cause of the small bowel obstruction was a 3 cm large enterolith impacted in the distal ileum (white arrows on Figure 3A – sagittal and B – axial views). A Meckel’s diverticulum was also incidentally discovered on the antimesenteric border of the ileum (black arrow on Figure 3C). Small bowel obstruction caused by the release of an enterolith from jejunal diverticulitis in the context of Diffuse Jejuno-Ileal Diverticulosis (DJID) was the final radiological diagnosis.

At the time of laparotomy, the enterolith had migrated further than the Meckel’s diverticulum, causing opportune dilatation of this diverticulum. Surgeons then performed an elective resection of the Meckel’s diverticulum wherein the enterolith could be manually and proximally retrogradely pushed. The inflamed jejunal diverticulum was also resected. The post-surgical period was uneventful.

## Comment

DJID incidence is about 0.06–2.3% in small bowel series and 0.3–4.5% in autopsy studies. This condition is twice more frequent in males; 80% of jejuno-ileal diverticula occur on the jejunum, 15% on the ileum, and 5% are located on both jejunum and ileum. Jejuno-ileal diverticula are acquired and pulsion type diverticula: they result from the herniation of the mucosa and submucosa through the muscle intestinal layer. They usually occur on the mesenteric border, at the entry point of mesenteric vessels, where the muscle layer is weak. Etiology of DJID is unclear but is probably linked to impaired intestinal smooth muscle contraction.

DJID can be clinically silent. Patients can also present non specific symptoms (60% of DJID) such as abdominal discomfort, early satiety and bloating. Steatorrhea and megaloblastic anemia due to vitamin B12 malabsorption are described as chronic complications due to stasis and bacterial overgrowth within the diverticula. The occurrence of acute complications is about 10–30% and include diverticulitis (consecutive to bowel stasis in a diverticulum, mucosal edema causing obstruction of diverticulum’s orifice and bacterial proliferation), perforation, intestinal hemorrhage and obstruction.

When DJID is incidentally discovered and remains asymptomatic, no surgical treatment is required. If chronic symptoms are present, conservative treatment should be tried before considering surgery. When complications occur, surgery may be considered (resection of affected intestinal segment and primary anastomosis is the current treatment).

Our clinical case combines diverticulitis and obstruction in a patient with DJID. We believe the initial event was the diverticulitis, which led to the expulsion of an enterolith. The escaped enterolith migrated in the intestinal tract and finally impacted in the ileum, causing obstruction.
